# A case of linear and whorled nevoid hypermelanosis

**DOI:** 10.7555/JBR.39.20250084

**Published:** 2025-06-25

**Authors:** Jinxu Qi, Yijia He, Guoqiang Zhang

**Affiliations:** 1 Department of Dermatology, the First Hospital of Hebei Medical University, Shijiazhuang, Hebei 050031, China; 2 Subcenter of National Clinical Research Center for Skin and Immune Diseases, the First Hospital of Hebei Medical University, Shijiazhuang, Hebei 050031, China; 3 Hebei Technical Innovation Center of Dermatology and Medical Cosmetology Technology, the First Hospital of Hebei Medical University, Shijiazhuang, Hebei 050031, China

Dear Editor,

Linear and whorled nevoid hypermelanosis (LWNH) is a rare, sporadic pigmentary disorder characterized by hyperpigmented macules arranged in linear streaks and whorls along Blaschko's lines, typically appearing within the first few weeks of life^[1]^, and remains a challenge to treat. Here, we report a case of LWNH and review the relevant literature to help clinicians better understand this disease.

The patient was a 2-year-old male. The primary presenting complaint was "hyperpigmented patches on the left trunk that had persisted for two years". The child was found to have linear hyperpigmentation on the left trunk since birth, and the lesions gradually expanded and increased in size. He had no erythema, blisters, or verrucous lesions during the course of the disease, reported no subjective symptoms, and had received no previous diagnosis or treatment. He was in good health; his parents were healthy, and there were no similar conditions in his family.

**Figure 1:**
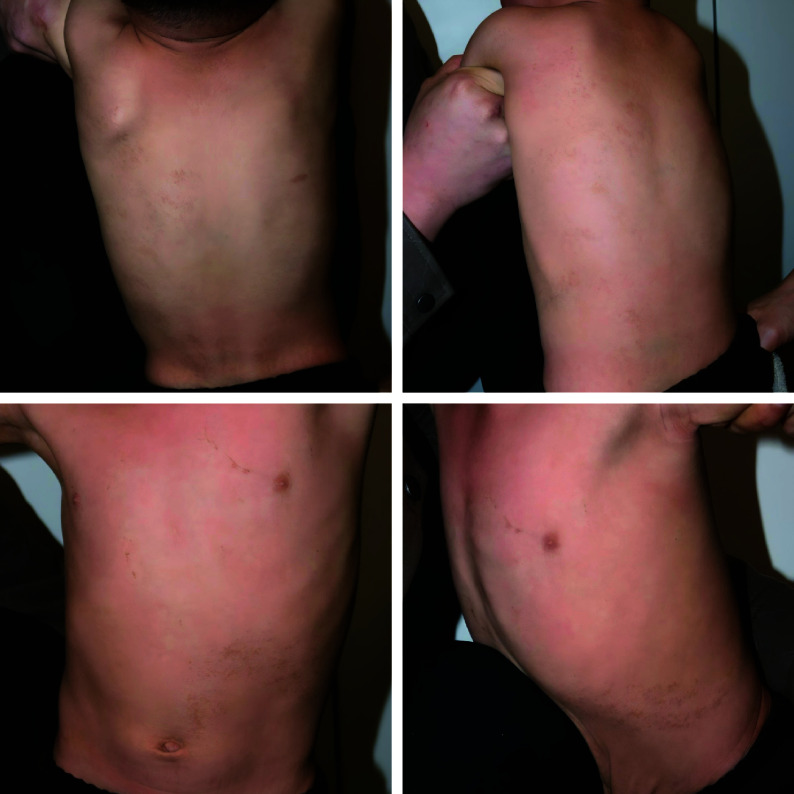
Dermatological examination of the patient. Hyperpigmentation of the left trunk in the form of lines, bands, and whorls, asymmetrically distributed along Blaschko's lines.

**Figure 2:**
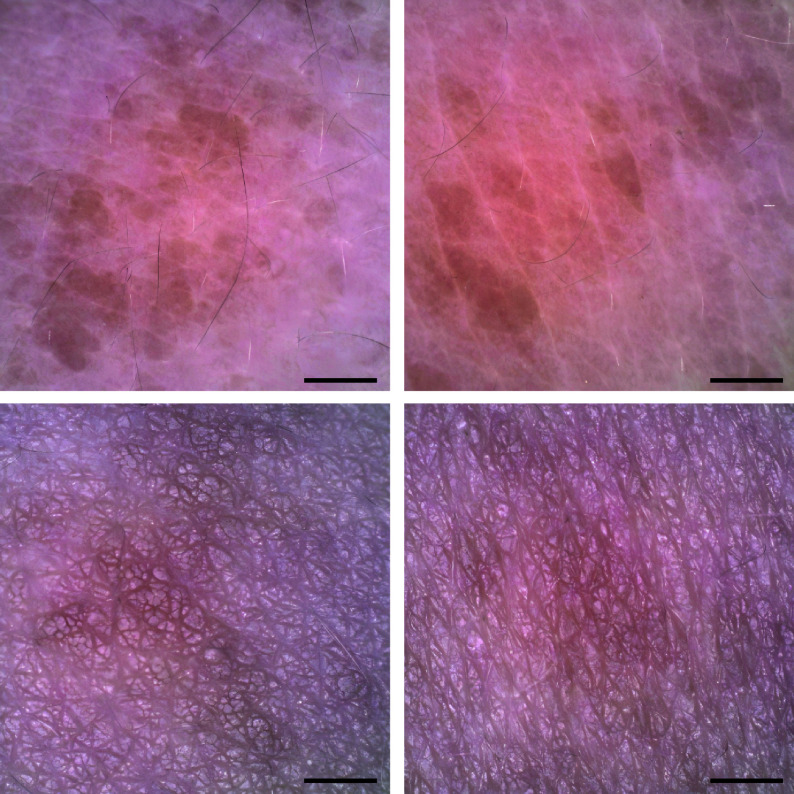
Dermoscopy of the patient. Brown patches arranged in lines. Scale bar, 250 μm.

Physical examination revealed normal development, normal intelligence, and no abnormalities in systemic examination. In particular, there were no abnormalities in the skeletal, cardiovascular, or central nervous systems, such as a depressed nasal bridge, a high-arched palate, atrial septal and ventricular septal defects, patent ductus arteriosus, tetralogy of Fallot, or epilepsy. No abnormalities were found on cardiac or abdominal ultrasonography. Dermatological examination revealed hyperpigmentation of the left trunk in the form of lines, bands, and whorls, asymmetrically distributed along Blaschko's lines (***Fig. 1***). Dermoscopy showed brown patches arranged in lines (***Fig. 2***). Final diagnosis: LWNH. His parents refused a histopathologic examination, and the child was placed under conservative observation. Intervention in LWNH is indicated when a patient has uncomfortable symptoms, rapidly progressing lesions, developmental delays, neurological or cardiovascular abnormalities, or a strong desire for treatment or cosmetic improvement.

LWNH is primarily associated with genetic mosaicism, which typically arises from somatic mutations during early embryonic development. This results in the proliferation and migration of two melanocyte lineages with different pigmentation potentials, leading to melanocyte dysfunction and subsequent hyperpigmented macules and whorls along Blaschko's lines^[2–4]^. The histopathological features of LWNH include an increase in melanin in the basal layer of the pigmented areas, without melanin incontinence.

LWNH should be clinically distinguished from incontinentia pigmenti, verrucous nevus, linear atrophoderma of Moulin (LAM), and progressive cribriform zosteriform hyperpigmentation (PCZH). Incontinentia pigmenti is an X-linked dominant disorder that is almost exclusively found in females, and most patients exhibit erythema and blisters before the onset of hyperpigmentation^[4–5]^. Verrucous nevus also appears as hyperpigmented streaks along Blaschko's lines in the early stages, but it develops papillomatous hyperplasia and hyperkeratosis over time^[5]^. LAM presents as hyperpigmented atrophic plaques, typically linear or banded, along Blaschko's lines; most patients exhibit no inflammatory reaction before the onset of lesions. The onset of LAM is slow, and it is self-limiting^[6]^. PCZH presents asymptomatic cribriform macular pigmentation distributed along Blaschko's lines. The mean age of PCZH onset is 14 years according to one series^[7]^, and it represents a late-onset and localized form of LWNH^[8]^.

Treating LWNH remains a challenge. Some researchers have attempted chemical peels and 2% hydroquinone^[6]^, narrowband ultraviolet B, and topical corticosteroids for treatment^[9]^, but these approaches have not yielded significant results. Catherine *et al*^[10]^ reported that Q-switched 532 nm and Q-switched 755 nm lasers were effective for the treatment of LWNH, while Q-switched 1064 nm laser treatment was ineffective. In another report, three Q-switched 694 nm laser treatments in a 4-year-old male patient resulted in a significant improvement of the lesion without recurrence^[11]^. This suggests that laser therapy may be the most effective method for treating LWNH, though careful selection of appropriate parameters is required. However, a major limitation is the small sample size, and larger case series or controlled clinical trials are needed in the future to confirm the most effective treatment for this disease.

Yours sincerely,

Jinxu Qi^1,2,3,△^, Yijia He^1,2,3,△^, Guoqiang Zhang^1,2,3,✉^

^1^Department of Dermatology, the First Hospital of Hebei Medical University,

Shijiazhuang, Hebei 050031,

China;

^2^Subcenter of National Clinical Research Center for Skin and Immune Diseases, the First Hospital of Hebei Medical University,

Shijiazhuang, Hebei 050031,

China;

^3^Hebei Technical Innovation Center of Dermatology and Medical Cosmetology Technology, the First Hospital of Hebei Medical University,

Shijiazhuang, Hebei 050031,

China.

^△^These authors contributed equally to this work.

^✉^Corresponding author: Guoqiang Zhang. E-mail: 57702800@hebmu.edu.cn.
